# Pneumatically-Actuated Acoustic Metamaterials Based on Helmholtz Resonators

**DOI:** 10.3390/ma13061456

**Published:** 2020-03-23

**Authors:** Reza Hedayati, Sandhya Lakshmanan

**Affiliations:** Novel Aerospace Materials group, Faculty of Aerospace Engineering, Delft University of Technology (TU Delft), Kluyverweg 1, 2629 HS Delft, The Netherlands; S.PazhayannurLakshmanan@student.tudelft.nl

**Keywords:** active noise control, acoustic metamaterial, broadband noise attenuation, pneumatic actuation, Helmholtz resonators

## Abstract

Metamaterials are periodic structures which offer physical properties not found in nature. Particularly, acoustic metamaterials can manipulate sound and elastic waves both spatially and spectrally in unpreceded ways. Acoustic metamaterials can generate arbitrary acoustic bandgaps by scattering sound waves, which is a superior property for insulation properties. In this study, one dimension of the resonators (depth of cavity) was altered by means of a pneumatic actuation system. To this end, metamaterial slabs were additively manufactured and connected to a proportional pressure control unit. The noise reduction performance of active acoustic metamaterials in closed- and open-space configurations was measured in different control conditions. The pneumatic actuation system was used to vary the pressure behind pistons inside each cell of the metamaterial, and as a result to vary the cavity depth of each unit cell. Two pressures were considered, P = 0.05 bar, which led to higher depth of the cavities, and P = 0.15 bar, which resulted in lower depth of cavities. The results showed that by changing the pressure from P = 0.05 (high cavity depth) to P = 0.15 (low cavity depth), the acoustic bandgap can be shifted from a frequency band of 150–350 Hz to a frequency band of 300–600 Hz. The pneumatically-actuated acoustical metamaterial gave a peak attenuation of 20 dB (at 500 Hz) in the closed system and 15 dB (at 500 Hz) in the open system. A step forward would be to tune different unit cells of the metamaterial with different pressure levels (and therefore different cavity depths) in order to target a broader range of frequencies.

## 1. Introduction

Metamaterials are periodic designer materials which offer physical properties not found in nature [1]. Particularly, acoustic metamaterials can manipulate sound and elastic waves both spatially and spectrally in unpreceded ways [2]. Such properties include super-focusing [3], super-lensing [4], active membrane structures [5,6], cloaking [7,8], phononic plates [9], fluid cavities separated by piezoelectric boundaries [10], and tunable noise attenuation based on Helmholtz resonators [11,12,13,14,15]. The capability of metamaterials to tune their physical behavior just based on their geometrical characteristics offers a great benefit over conventional materials for application in various high-demand industries such as aerospace, automobile, and construction. Increase in importance of customer comfort in transportation and construction sectors as well as consumer goods has been a driving force for recent advancements in acoustic metamaterials in the field of noise control. Acoustic metamaterials can generate arbitrary acoustic bandgaps by scattering sound waves, which is a superior property for insulation properties.

Initially, the acoustic metamaterials were passive; their performance remained unchanged and it was in accordance to the design values [16,17,18]. More recently, the concept of tunable acoustic metamaterials has emerged in which acoustic metamaterials are able to manipulate sound waves in various conditions [6,7,8,19,20,21]. Active acoustic metamaterials can be responsive to different stimuli, such as electric field [21], electromagnetic field [6,22], mechanical load [11], and electricity by means of piezoelectric elements [7]. 

**Figure 1:**
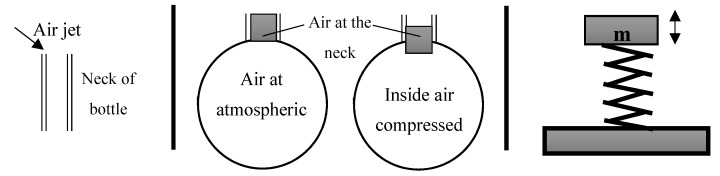
Phenomenon of Helmholtz resonance where air inside the cavity is modelled as a spring mass system (redrawn from [24])**.**

Acoustic metamaterials are widely preferred in applications where high efficiency in noise reduction is expected. As an important design parameter in this regard is simplicity in design yet being effective in performance, Helmholtz resonance provides a lucrative solution (Figure 1). Due to its simplistic geometrical characteristics, Helmholtz resonators have been incorporated into acoustic metamaterials. The Helmholtz resonator is a phenomenon where acoustic resonance is created when a gas (typically air) passes over the opening (also called neck) of an enclosed cavity. The system working resembles that of a mass-spring system. The air inside the cavity works as a spring and the air in the neck works as a mass. When an incoming air pressure acts upon the air occupying the neck region, it pushes it down into the cavity. Hence, the air inside the cavity becomes compressed and attempts to retain its original volume. This pushes back the air inside the neck and creates a counteracting acoustic resonance with respect to the initial incoming acoustic wave. When the air pressure inside the cavity decreases, it sucks air around the neck area back again, and this phenomenon repeats itself several times making the air inside the cavity work similar to a spring. In recent years, there has been extensive research in development of acoustic metamaterials constructed from Helmholtz resonator unit cells [13,14,15] to generate tunable band gaps.

The Helmholtz resonator equation is [23]

(1) $$f=\frac{c}{2\pi }\sqrt{\frac{A}{VL}}$$

where c is the speed of sound in the medium (i.e., air), L is the neck length, A is the neck cross-sectional area, and V is the volume of medium in the cavity. As it can be deduced from Equation (1), three geometrical characteristics (cross-sectional area A and length L of neck as well as the volume of the cavity V) can be actively altered to produce tunability in the performance of metamaterial. The most convenient way to actively adjust the performance of metamaterial is tuning the cavity volume as the actuation system would not need to perform in the vicinity of the neck where the metamaterial interacts the most with incoming external sound pressures.

Sui et al. [25] designed and tested light-weight yet sound-proof acoustic metamaetrials made of hexagonal honeycomb structure covered by a rubbery memberane sheet. Covering the sandwich panel with the membrane resulted in an increased sound transmission loss up to 25 dB at f = 200 Hz. In another honeycomb-based structure, Claeys et al. [26] introduced a vibro-acoustic acoustic metamaterial in which they had implemented resonant elements onto the unit cell of a rectangular core structure. They investigated the most influencing parameters of their passive metamaterial using numerical and experimental methods. The relative mass of the added resonant structure to the host structure was found to be the most influencing parameter on the noise attenuation.

As one of the first examples of adaptive acoustic systems based on Helmholtz resonators, Estève and Johnson [27] implemented a motorized iris diaphragm in the neck region of a simply supported cylinder (56 cm in length and 12.7 cm in diameter) to attenuate noise. They externally altered the neck opening diameter from 9 to 58 mm, which led to narrow-band noise reduction of maximum 8 dB in frequencies of 63, 82, and 103 Hz.

Different types of actuation systems have been explored in previous studies for altering various dimensional parameters of active Helmholtz resonators (see Table 1). Birdsong and Radcliffe [28] designed a tunable semi-active Helmholtz resonator (SHR) that was able to reduce the resonance frequency and peak magnitude of noise in the frequency range of 60–180 Hz effectively. Their system was designed to be most effective in reducing noise in primary acoustic systems, such as ducts or pipes, by actively changing the cavity depth using electronic actuators. Bedout et al. [29] proposed another tunable resonator design that operated on the basis of radially blocking parts of cylindrical cavity volume by means of a rotary-actuated moving plate. Their design demonstrated up to 29 dB noise reduction in the frequency range of 65–150 Hz. Nagaya et al. [30] introduced a rotary two-stage Helmholtz resonator that had auto-tuning control capabilities at both low (100–3000 Hz) and high frequencies (1000–3000 Hz) for application in typical blowers.

Due to reasons mentioned above, the most logical way to control the dimensions of a Helmholtz resonator in an active system would be to change the volume of the resonator cavity. In this study, this method is realized by altering one dimension of the resonators (depth of cavity) by means of pneumatic actuation system. To this end, metamaterial slabs are additively manufactured and connected to a proportional pressure control unit. The noise reduction performance of active acoustic metamaterials in closed- and open-space configurations are measured in different control conditions.

## 2. Materials and Methods

### 2.1. System Design

**Figure 2:**
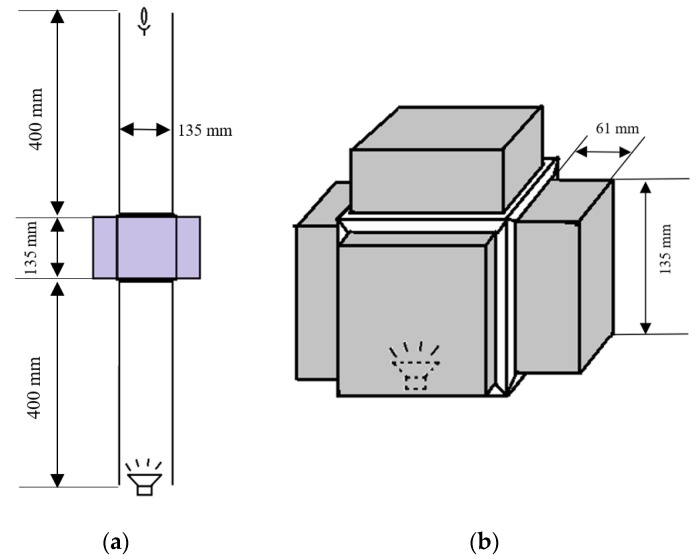
(**a**) Open and (**b**) closed systems design to measure the performance of active acoustic metamaterials.

Two types of systems were considered for acoustic measurements: an open system and a closed system (Figure 2). The closed system consisted of a cube, 5 walls of which was made up of 8 × 8 arrays of unit cells together constructing a plate metamaterial. Each unit cell had a cavity with a depth of 42 mm and a cross-section of 14 × 14 mm^2^ and a neck of diameter of 1.2 mm and length of 14 mm. A speaker was placed in the middle of the system, and the noise mitigation was measured outside the cube and 15 cm away from the walls of the cube. The open system composed of two long aluminum channels with square cross-section linked together via a 4-walled cubic metamaterial in the center. A speaker was placed at one side of the main channel and the noise was measured at the other end of the tube. The noise attenuation levels were measured with a microphone for different frequencies to determine the performance of the metamaterials at different frequencies. A Sennheiser e908B cardioid condenser microphone (frequency response of 40–20,000 Hz, Sennheiser, Wedemark, Germany) was used as the recording instrument. The sound pressure levels (SPL) were obtained by taking a fast Fourier transform (FFT) of the recorded sound clips, thus changing the output from a time domain plot to a frequency domain plot. These plots were normalized with respect to a reference ambient pressure plot.

Four types of noise samples, namely white noise, pink noise, brown noise, and a frequency sweep of 10,000→20 Hz were used for the acoustic tests. A tone generator application was used to produce each of the noted noise samples. The difference between colors of noise used in this study is described below [11]:

- White noise: white noise has a uniform signal power intensity distribution at all frequencies when frequency is plotted in Hz scale, giving a uniform power spectral density.
- Pink noise: In homogeny with white noise, the pink noise has a uniform power distribution if the bandwidth is plotted in a logarithmic scale. Consequently, if the frequency spectrum is plotted linearly, the sound magnitude is mainly concentrated at the lower end of the spectrum.
- Brown noise: In brown noise, the power amplitude decreases with a proportion of $1/f^{2}$, with respect to frequency. In other words, in brown noise sample, the power amplitude decreases 6 dB per octave.
- A frequency sweep of 10,000→20 Hz to provide a diverse range of narrow-band frequency tones while measuring loudness in real time.

### 2.2. Manufacturing

**Figure 3:**
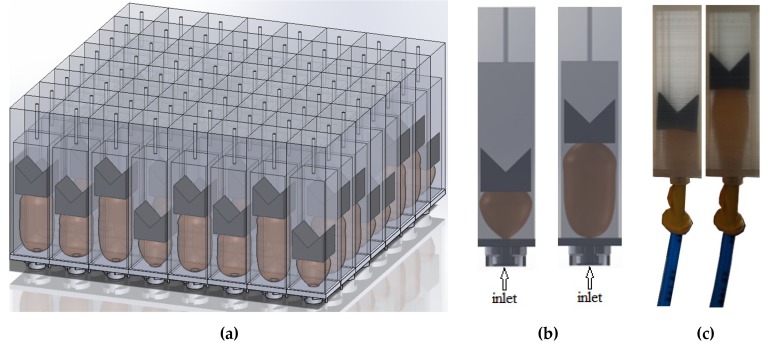
(**a**) Schematic of a metamaterial slab equipped with actuating balloons; (**b**) schematic of variation of the cavity depths with balloons in the deflated and inflated states; (**c**) image of actuation of a 3D-printed unit cell in the deflated and inflated states.

**Figure 4:**
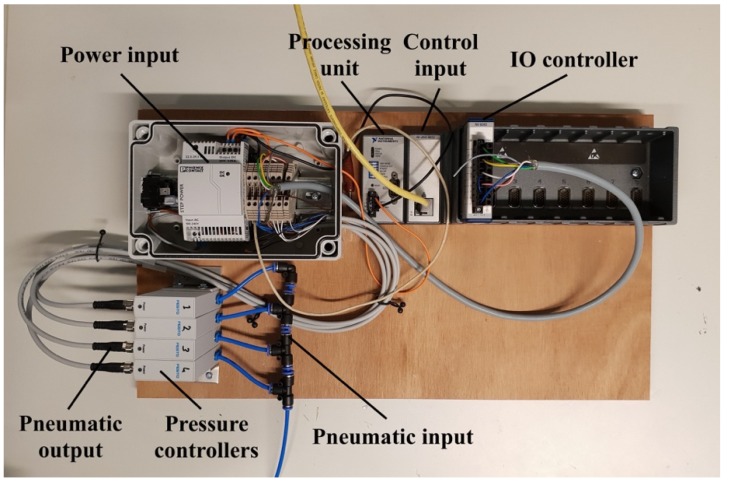
The pneumatic actuation system used for inflating and deflating balloons in the active acoustic metamaterial.

A metamaterial slab equipped with pneumatic actuation is demonstrated in Figure 3. In theory, each individual unit cell has the capability of having a different cavity depth. Each cavity has a piston the location of which can be tuned through varying the pressure of an inflatable balloon under it (Figure 4). The metamaterial walls, pistons were additively manufactured using Fused Deposition Modeling (FDM) additive manufacturing (AM) technique. Polylactic acid (PLA) was chosen as the printing material, and Ultimaker 2+ and Ultimaker 3 3D printer apparatus (Untilmaker, Utrecht, Netherlands) were used. To establish a comparative device for the performance of the metamaterial, a solid plate of similar mass (430 g) was manufactured using the same manufacturing technique and settings and used a reference case. Such cases will be named as “plain isolators” in the remaining of the manuscript.

Balloons create the minimum and maximum depths at respectively their inflated and deflated status. The only drawback of this mechanism is that the accuracy of depths of the cavities is in millimeter range, as particular air pressures can lead to slightly different balloon inflation in different unit cells depending on the friction between the piston and inner walls of each unit cell. 

### 2.3. Actuation

**Figure 5:**
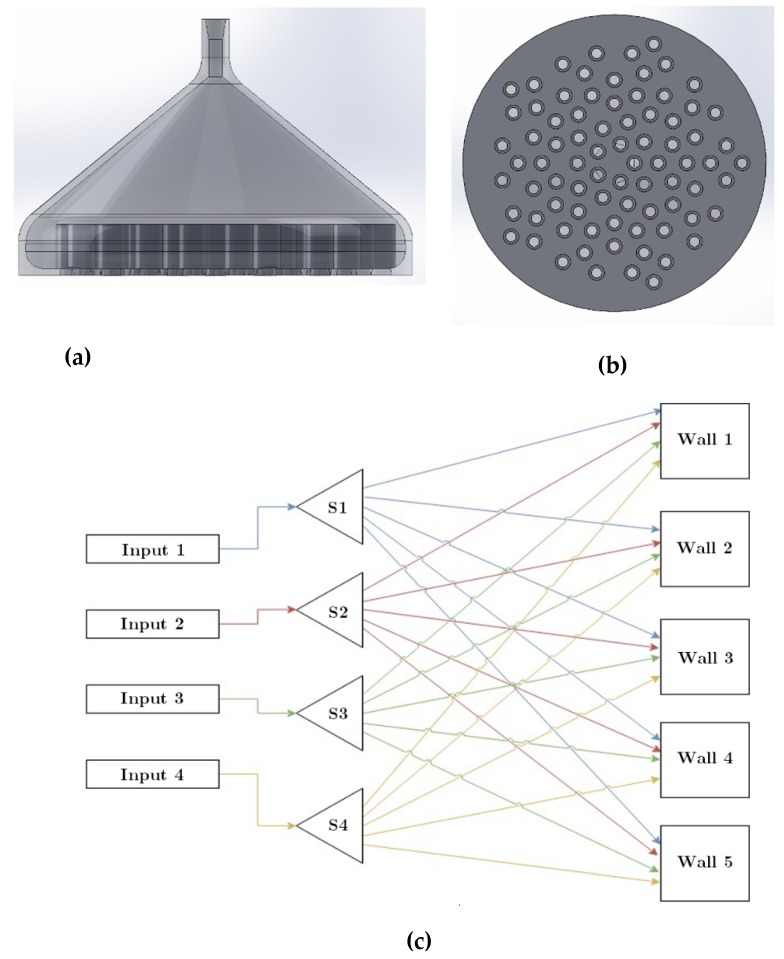
(**a**) Side and (**b**) bottom views of the splitter. (**c**) Drawing of the connection from the pressure outlet of the pneumatic system to the splitters (S1, S2, S3, and S4) and then distributed to the unit cells of the metamaterial slabs (walls) in a closed-space system. Each line color represents the connection of each splitter to different cells of metamaterial slabs.

The pneumatic inlets of each balloon are connected to a digital pressure regulator controlled by a house-made software made by LabView. The pressure controller consists of different units such as power unit, processing unit, Input/output (IO) controller, and four proportional pressure controllers (VEAB-L-26-D9-Q4-V1-1R1, Festo, Delft, The Netherlands), see Figure 4. Since the number of proportional pressure controllers is limited to four, a splitter (Figure 5a,b) is manufactured for the outlet of each pressure controller so that pressure provided by the pressure controller can be transferred to several unit cells of metamaterial (Figure 5c). The closed setup is composed of five metamaterial walls each consisting of 64 resonators, and hence 80 outlets are drawn from each pressure splitter.

**Figure 6:**
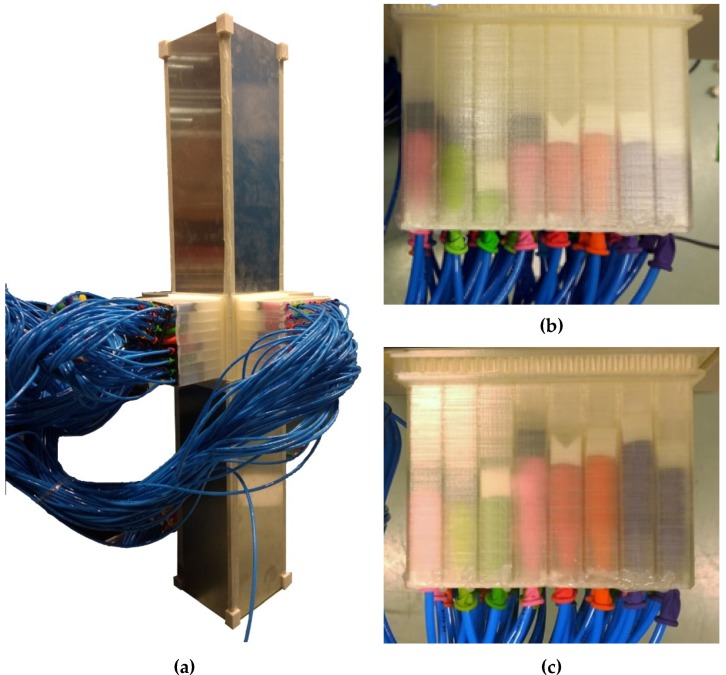
(**a**) The 3D-printed metamaterial in the open configuration with tubes connecting the inflatable balloons in each unit cell to the splitters of the pneumatic actuation, (**b**) resonators at P = 0.05 bar, (**c**) resonators at P = 0.15 bar.

The pressure outlets of the pneumatic system (Figure 4) are connected to the balloons fixed inside the resonators by means of flexible tubes. The balloons are attached to the back side of each unit cell, and each resonator cavity is sealed as the balloons are glued to the back side of the pistons. Figure 6 displays how pressure is distributed from the pneumatic pressure provider system to different resonators by means of four different splitters. Furthermore, two different cavity depths obtained by pressures of 0.05 bar and 0.15 bar are demonstrated. The metamaterial is tested at two pressures; the pressure 0.05 bar yields the highest possible cavity depths, while 0.15 bar yields the lowest cavity depth. As the open system is composed of only four metamaterial slabs (Figure 2a), the remaining 64 outlets of the splitter are sealed.

## 3. Results and Discussions

In each input pressure level, due to the non-identical response of the inflatable balloons to the pressure level, the cells had a range of cavity depths (with variations of ~3 mm) rather than a single cavity depth. The number of acoustic tests conducted was limited as the fatigue endurance of commercially available balloons is relatively low, allowing only for a limited number of actuations.

### 3.1. Open System

**Figure 7:**
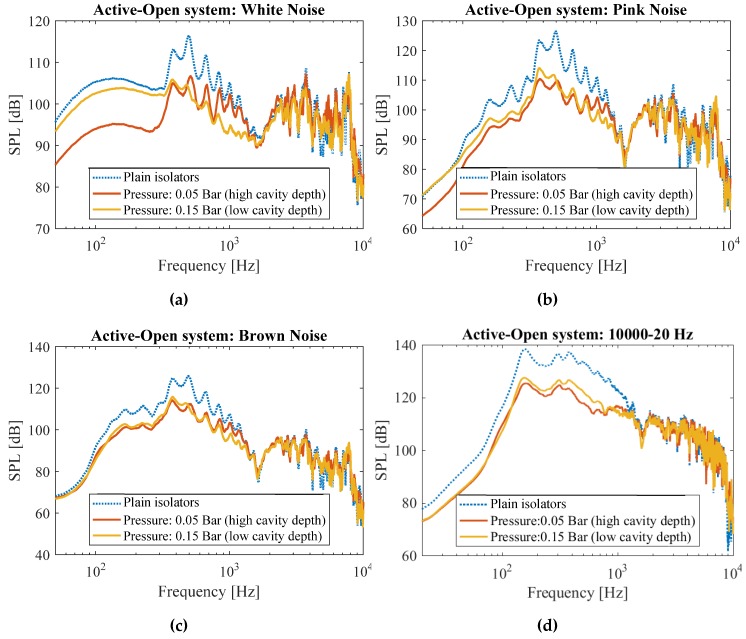
Measured sound pressure levels in the open system for (**a**) white, (**b**) pink, (**c**) brown noises, as well as for (**d**) frequency sweep from 10,000→20 Hz for systems with plain isolators, and the active system at two different cavity depth levels.

Decreasing the input pressure from 0.15 bar to 0.05 bar (and therefore increasing the cavity depth) improved the noise mitigation in low-frequency range, particularly for white noise and pink noise. For instance, for the case of white noise, at a frequency of 100 Hz, the metamaterial with higher depth (P = 0.05 bar) has 9 dB more noise mitigation as compared to the metamaterial with lower depth (P = 0.15 bar), see Figure 7. Similarly, for the case of pink noise, the metamaterial with higher depth (P = 0.05 bar) has 3.5 dB more noise mitigation as compared to the metamaterial with lower depth (P = 0.15 bar), see Figure 7a.

In high frequencies, on the other hand, the metamaterial with lower depth (P = 0.15 bar) demonstrates better performance as compared to the metamaterial with higher depth (P = 0.05 bar). For instance, for the case of white noise, at a frequency of 1000 Hz, the metamaterial with higher depth (P = 0.05 bar) has 5 dB more noise mitigation as compared to the metamaterial with lower depth (P = 0.15 bar), see Figure 7. Similarly, for the case of pink noise, the metamaterial with higher depth (P = 0.05 bar) has 3 dB more noise mitigation as compared to the metamaterial with lower depth (P = 0.15 bar), see Figure 7b.

The difference between the performance of both systems became negligible for the cases of brown noise and frequency sweep 10,000→20 Hz, see Figure 7c,d. However, both the systems are able to mitigate noise for around 10 dB for frequency range of 100 Hz to 1000 Hz (Figure 7c,d).

In summary, the system with high cavity volumes (P = 0.05 bar) demonstrated a better performance at lower frequencies, while the system with low cavity volumes (P = 0.15 bar) reduced the noise more efficiently at the higher end of the frequency spectrum (Figure 7). This was an expected performance from the active metamaterial, since according to the Helmholtz resonance equation (i.e., Equation (1)) where $f\propto V^{-\frac{1}{2}}$.

### 3.2. Closed System

**Figure 8:**
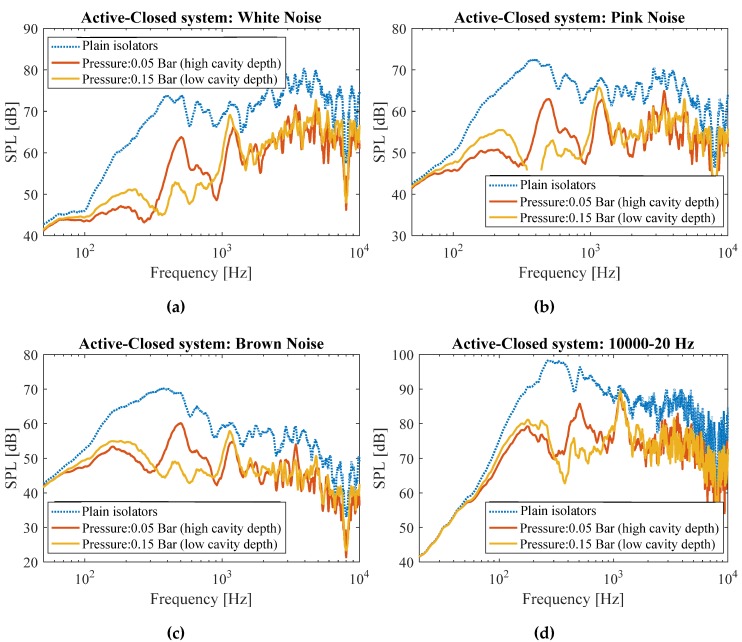
Measured sound pressure levels in the open system for (**a**) white, (**b**) pink, (**c**) brown noises, as well as for (**d**) frequency sweep from 10,000→20 Hz for systems with plain isolators, and the active system at two different cavity depth levels.

Similar to open system, in the lower end of frequency range, the system performed better when it was at its high cavity depth state (P = 0.05 bar), and the system performed better in higher frequencies when the cavity depth was lower (P = 0.15 bar), see Figure 8. The maximum noise mitigation of high-cavity-depth system as compared to low-cavity-depth system was observed at f = 200 Hz for all the noise sample types (white, pink, brown, and frequency sweep 10,000→20 Hz), in which the high-cavity-depth system mitigated noise 3–4 dB better than its low-cavity-depth counterpart. On the other hand, the maximum noise mitigation of low-cavity-depth system as compared to high-cavity-depth system was observed at f = 500 Hz for all the noise sample types (white, pink, brown, and frequency sweep 10,000→20 Hz), in which the low-cavity-depth system mitigated noise 8–10 dB better than its high-cavity-depth counterpart (Figure 8).

Overall, making use of metamaterial for the closed system led to 8–20 dB noise mitigation as compared to the system without metamaterial (composed of solid walls) in the frequencies higher than 100 Hz.

In another study [11], we used a stepper motor to vary the depth of the cavities. As compared to linear motor, the pneumatic mode of actuation has both advantages and disadvantages. In the pneumatically-actuated system, a much smaller number of actuators (i.e., linear pressure providers) are needed to create non-uniform distribution of cavity depths, whereas for the case of actuation by means of the linear motor, theoretically, each unit cell requires a dedicated linear actuator. Therefore, the linear motor actuation mechanism has much more limitations regarding individual depth of cavities in each metamaterial slab, if the economic point of view is to be considered. On the other hand, linear actuators provide a better precision in resonator location.

## 4. Conclusions

In this study, pneumatically-actuated acoustic metamaterials based on Helmholtz resonator were developed and their acoustic performance was analyzed. The pneumatic actuation system was used to vary the pressure behind pistons inside each cell of the metamaterial, and as a result to vary the cavity depth of each unit cell. The results showed that by increasing the pressure level, the mitigation region of performance shifts from a frequency range of 150–350 Hz at higher cavity depths to a frequency range of 300–600 Hz at a lower cavity depth. The pneumatically-actuated acoustical metamaterial yielded peak attenuations of 20 dB and 15 dB in respectively closed and open systems (at 500 Hz). The results of the study demonstrate that pneumatic actuation system can be effectively implemented to mitigate a targeted range of frequencies for noise reduction applications in low frequencies such as in aircraft liners, buildings, and noise mitigation enclosures. A step forward would be to tune different unit cells of the metamaterial with different pressure levels (and therefore different cavity depths) in order to target a broader range of frequencies.

## Figures and Tables

**Table 1:** Comparison of performance and actuation technique of metamaterials based on Helmholtz resonators.

Type of Volume Change (neck/cell).	Decrease in Noise (dB)	Range of Frequency (Hz)	Actuation Type	Range of the Changing Parameter	Type (self-Tuning, Active, Adjustable)	Ref.
Cavity length	100%	80–170	Pneumatic	N/A	Adjustable	[28]
Neck diameter	4.2 dB	75–115	Rotary-actuated aperture	9–58 mm	Adjustable	[27]
Volume (Sector angle change)	29 dB	65–150	Rotary-actuated walls	1491–14,093 cm^3^	Self-tuning	[29]
Cavity length	20 dB	-	Hydraulic	43–243 mm	Active	[31]
Internal pressure	-	50–500 (Max-160)	Electromagnetic diaphragm/piezoelectric	N/A	Active	[32]
Cavity length	18 dB	0–400 (Max-226)	-	60–90 mm	Adjustable	[33]
Neck length	-	3–75	Hydraulic	39–60 cm	Adjustable	[34]
Length of cavity	30 dB	80–140	Pneumatic	560–940 cm^3^ 1.8–17 cm	Adjustable	[35]
Area of neck	25 dB	100–3000	Rotary	-	Self-tuning	[30]
